# Advanced glycation end products and reactive oxygen species: uncovering the potential role of ferroptosis in diabetic complications

**DOI:** 10.1186/s10020-024-00905-9

**Published:** 2024-09-09

**Authors:** Yanchi Chen, Zihan Meng, Yong Li, Shibo Liu, Pei Hu, En Luo

**Affiliations:** 1 State Key Laboratory of Oral Diseases & National Center for Stomatology & National Clinical Research Center for Oral Diseases, West China Hospital of Stomatology, Sichuan University, Chengdu, 610041 Sichuan China; 2 State Key Laboratory of Oral Diseases & National Center for Stomatology & National Clinical Research Center for Oral Diseases & Department of Oral Maxillofacial Surgery, West China Hospital of Stomatology, Sichuan University, Chengdu, 610041 Sichuan China

**Keywords:** Advanced glycation end products, Oxidative stress, Ferroptosis, Diabetic complications

## Abstract

Advanced glycation end products (AGEs) are a diverse range of compounds that are formed when free amino groups of proteins, lipids, and nucleic acids are carbonylated by reactive carbonyl species or glycosylated by reducing sugars. Hyperglycemia in patients with diabetes can cause an overabundance of AGEs. Excess AGEs are generally acknowledged as major contributing factors to the development of diabetic complications because of their ability to break down the extracellular matrix directly and initiate intracellular signaling pathways by binding to the receptor for advanced glycation end products (RAGE). Inflammation and oxidative stress are the two most well-defined pathophysiological states induced by the AGE–RAGE interaction. In addition to oxidative stress, AGEs can also inhibit antioxidative systems and disturb iron homeostasis, all of which may induce ferroptosis. Ferroptosis is a newly identified contributor to diabetic complications. This review outlines the formation of AGEs in individuals with diabetes, explores the oxidative damage resulting from downstream reactions of the AGE-RAGE axis, and proposes a novel connection between AGEs and the ferroptosis pathway. This study introduces the concept of a vicious cycle involving AGEs, oxidative stress, and ferroptosis in the development of diabetic complications.

## Background

Diabetes is a collection of metabolic conditions distinguished by high blood glucose levels arising from impairments in insulin secretion, insulin activity, or both (American Diabetes Association 2011). The increasing prevalence of diabetes and its complications remains an enormous burden globally. The prevalence of diabetes worldwide has been increasing in recent years, with an anticipated increase to 1.31 billion cases by 2050 (GBD 2021 Diabetes Collaborators 2023). Moreover, diabetes is the eighth most common cause of death (GBD 2021 Diabetes Collaborators 2023). Previous studies have demonstrated that most patients diagnosed with diabetes experience at least one complication, and even patients with prediabetes have a significantly greater chance of developing complications in their kidneys, eyes, and cardiovascular system (Schwartz et al. 2017). The complications of diabetes strongly affect patient survival and quality of life; therefore, understanding the molecular mechanism of diabetes complications will improve the prognosis of this disease. However, the mechanisms underlying diabetes complications caused by hyperglycemia are still unclear.

Research has revealed a correlation between AGEs and the development of various complications associated with diabetes, such as diabetes nephropathy, diabetes retinopathy, and osteoporosis (Rabbani and Thornalley 2018; Yang et al. 2021; Kang and Yang 2020). AGEs are typically formed through a classic Maillard reaction, where reducing sugars react with the amino groups of proteins, lipids, and nucleic acids in a nonenzymatic manner. This process involves the formation of a Schiff base, followed by an Amadori rearrangement and oxidative modifications (glycoxidation). A hyperglycemic environment can promote the production of AGEs through various pathways (Khalid et al. 2022). The receptor for advanced glycation end products (RAGE) on the cell membrane plays a pivotal role in facilitating the intracellular pathogenic responses elicited by AGEs (Hecker et al. 2014). An abnormal increase in AGEs can produce a variety of downstream effects related to cell survival and function by binding with RAGE, which is extremely important for the occurrence of diabetes complications (He et al. 2022).

By blocking redox balance, AGEs can cause oxidative stress. This stress could be the source of ferroptosis, which is a novel mechanism of cell death. Unlike apoptosis and necrosis, ferroptosis is triggered by excessive phospholipid hydroperoxide accumulation in an iron-dependent manner (Yan et al. 2021). Recent studies have shown that the downstream consequences of AGEs are connected to the emergence of ferroptosis, which in turn contributes to tissue damage. This damage leads to complications of diabetes, such as diabetic cardiomyopathy, diabetes osteoporosis, diabetes nephropathy and diabetes peripheral neuropathy (Wang et al. 2022a, b; Kim et al. 2021a, b; Tang et al. 2022a, b; Ge et al. 2022). This review is the first to propose that AGEs serve as the origin of both oxidative stress and ferroptosis and that oxidative stress and ferroptosis byproducts coherently contribute to the formation of AGEs, creating a vicious cycle among AGEs, oxidative stress and ferroptosis. This proposed process may contribute to the development of diabetic complications.

## ROS promoted AGE production

### The mechanism of AGE formation

The formation of AGEs involves a dual-stage mechanism; the generation of reactive carbonyl species (RCSs) is followed by the carbonylation of proteins. RCSs, such as glyoxal, glyceraldehyde, glycolaldehyde, methylglyoxal, malondialdehyde, and 3-deoxyglucosone, can be derived from carbohydrate metabolism, glycation reactions, lipid peroxidation and protein oxidation (Ahmad et al. 2018; Tang et al. 2019). These processes are key to AGE formation and introduce carbonyl groups into the side chains of proteins, particularly nucleophilic lysine and arginine groups. These changes covalently modify protein structure and ultimately fuel the AGE pool (Singh et al. 2001; Hecker and Wagner 2018; Akagawa 2021). From a derivation perspective, AGEs can be classified into two types: advanced glycation end products (AGEs), which originate from carbohydrate-related pathways, and advanced lipoxidation end products (ALEs), which originate from lipid peroxidation (Ott et al. 2014).

AGE generation is intricate and involves the Maillard reaction, the polyol pathway, the Namiki pathway, the Wolff pathway, the Hodge pathway, glycolysis and ketone body metabolism (Khalid et al. 2022). The classic and predominant pathway among these pathways is the Maillard reaction, which can be divided into three distinct stages: early condensation, intermediate degradation and final production (Chuyen 2006). Initially, the carbonyl group from a reducing sugar, such as aldose or ketose, reacts nonenzymatically with the primary amino group from a protein, especially arginine and lysine, to yield the Schiff base (Zeng et al. 2019). Ketose (fructose) can also be derived from aldose (glucose) via the polyol pathway, with aldose reductase (AR) and sorbitol dehydrogenase (SDH) catalyzing the reaction (Clements 1986). The intermediate Schiff bases are unstable and reversible and undergo a subsequent rearrangement to form more stable aldoamines named Amadori products (Kim et al. 2017; Tsekovska et al. 2016). Ketoamines, the so-called Heyn products, are analogs of aldoamines in the polyol pathway (Tsekovska et al. 2016; Twarda-Clapa et al. 2022). As the cascade reaction progresses, the majority of the abovementioned processes lead to the production of important intermediates, such as RCS.

Nevertheless, the case is more severe regarding ALEs. Lipids, particularly membranous polyunsaturated fatty acyl tail-containing phospholipids (PUFA-PLs), are extremely vulnerable to reactive oxygen species (ROS), making them key targets of ROS attack (Singh et al. 2015). Excessive levels of ROS can oxidize PUFA-PLs through either the nonenzymatic Fenton reaction or the enzymatic pathway that uses labile free iron or iron-dependent enzymes to catalyze the reaction, respectively, yielding PUFA phospholipid hydroperoxides (PUFA-PL-OOHs). If the antioxidant system fails to promptly eliminate the initial PUFA-PL-OOHs, they further breakdown into alkoxyl and peroxyl radicals, leading to the cascading proliferation of PUFA-PL-OOHs. Finally, this chain reaction breaks down PUFA-PLs and produces a large amount of RCS. Malondialdehyde (MDA) and 4-hydroxynonenal (4-HNE), which are both components of RCS, are typically recognized as reliable indicators of the extent of lipid peroxidation (Wu et al. 2021a, b; Sun et al. 2022). The aforementioned mechanism by which RCSs are initiated through lipid peroxidation is also a component of the programmed cell death process of ferroptosis (Jiang et al. 2021; Stockwell 2022). Thus, ferroptosis may lead to an increase in the endogenous generation of AGEs.

While lipid peroxidation-derived RCSs are typically categorized as α,β-unsaturated-aldehydes, dialdehydes, and ketoaldehydes (Altomare et al. 2021), AGE-related RCSs are mostly saturated dicarbonyl compounds (Reddy et al. 2022). However, because of the close resemblance between AGEs and ALEs, both are referred to as AGEs in the following section.

### ROS are at the center of AGE elevation in diabetic conditions

One of the primary symptoms of diabetes is hyperglycemia, which is the main contributor to long-term diabetic complications (ElSayed et al. 2023). Because reducing sugars serve as the main substrate for the synthesis of AGEs, it is conceivable that high blood sugar levels could lead to the accumulation of AGEs. The level of AGEs in the urine possibly indicates that in vivo AGE levels are positively correlated with dietary glycemic load (Maasen et al. 2019). However, this process is complicated. Prolonged hyperglycemia increases the flux of glucose through several carbohydrate metabolic pathways, particularly glycolysis and the tricarboxylic acid cycle (TCA cycle), causing metabolic dysfunction (Lund et al. 2019). As a result of increased glycolytic activity and TCA cycle flux, NADH and FADH2 accumulate, which imposes a significant strain on the mitochondrial electron transport chain (Yan 2014). The interference of electron transfer can lead to the generation of superoxide rather than water by coenzyme Q, as it donates superfluous electrons to molecular oxygen (Kang and Yang 2020). Consequently, this can result in excessive production of ROS. The accumulation of NADH, superoxide and ROS can potentially impede the activity of glyceraldehyde-3-phosphate dehydrogenase (GAPDH), which is a fundamental component of glycolysis and is responsible for converting glyceraldehyde-3-phosphate into 1,3-biophosphoglycerate (Yan 2014; Yuan et al. 2019). During glycolysis itself, the inhibition of GAPDH results in the accumulation of glyceraldehyde-3-phosphate (GA-3-p) and its isomeric compound, dihydroxyacetone phosphate (DHAP); these triose phosphates then breakdown into methylglyoxal (MG) upon dephosphorylation (Ighodaro 2018). MG has been extensively studied as an RCS, and it serves as an important component for the production of AGEs. Additionally, MG has the ability to irreversibly inhibit the activation of GAPDH, leading to further suppression of the glycolysis pathway (Barinova et al. 2023).

Regarding carbohydrate metabolism, the glycolysis pathway is inhibited by GAPDH inactivation under hyperglycemic conditions; therefore, the increased amounts of glycolytic intermediates may be shunted to several subsidiary glucose-utilizing pathways, including the polyol pathway, the hexosamine pathway, and the protein kinase C (PKC) pathway (Kang and Yang 2020; Giacco and Brownlee 2010; Yumnamcha et al. 2020). In addition to being an AGE producer, as mentioned above, the polyol pathway is also a pro-ROS pathway. In the primary phase, AR facilitates the conversion of glucose to sorbitol, employing NADPH as the reducing agent. Excessive consumption of NADPH by the alternatively enhanced polyol pathway may cause a decrease in glutathione (GSH) synthesis. This decrease diminishes the antioxidative stress capacity and leads to oxidative stress (Abdelkader et al. 2022). The second step of this process involves SDH, which catalyzes the oxidation of sorbitol to fructose by converting the cofactor NAD + to NADH. A disproportionate NADH/NAD + ratio, accompanied by a substantial amount of NADH, increases mitochondrial ROS production (Kang and Yang 2020). In addition to the polyol pathway, ROS overproduction in diabetes mellitus is also attributed to the hexosamine and PKC pathways (Ighodaro 2018).

**Fig. 1:**
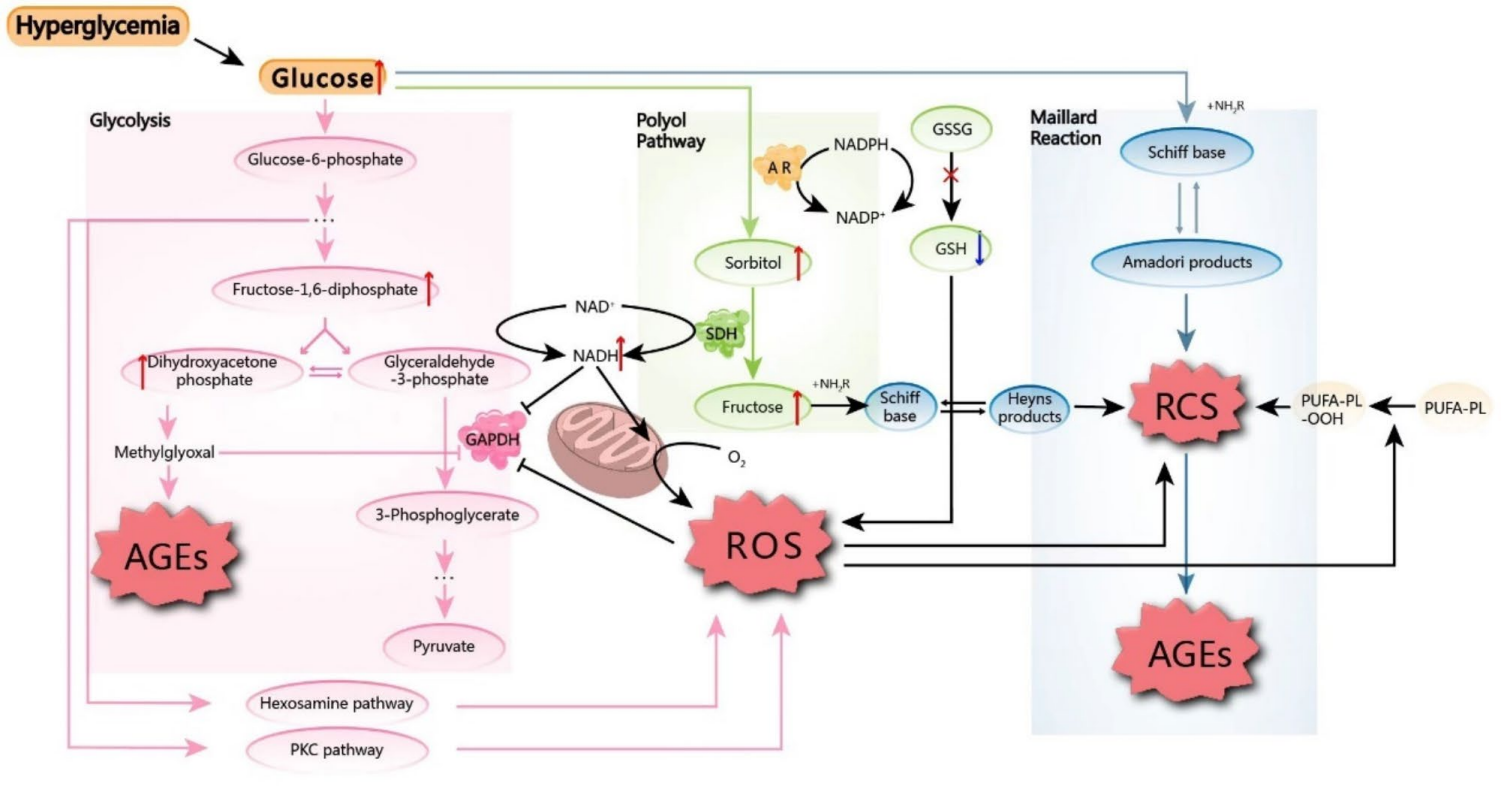
RCSs are important intermediates of AGE formation. These compounds are derived mainly from the Maillard reaction, which does not involve enzymes, or the polyol pathway, which involves AR and SDH. ROS may be strong catalysts for AGE formation. Hyperglycemia increases ROS levels via metabolic dysfunction, including alterations in glycolysis, the polyol pathway, the hexosamine pathway, and the protein kinase C (PKC) pathway. Notably, the inhibition of the fundamental protein GAPDH and the disruption of mitochondrial electron transfer chains are particularly significant. Excessive levels of ROS can attack PUFA-PLs, creating lipid peroxide-derived agents that contribute to the RCS pool. Moreover, the overproduction of ROS causes a redox imbalance, which directly gives rise to RCS

Excessive ROS can attack PUFA-PLs, creating lipid peroxide-derived agents that contribute to the RCS pool. Moreover, the overproduction of ROS causes a redox imbalance. This imbalance directly gives rise to RCS (Tian and Zhen 2019), which is a significant intermediate of AGE formation. Hence, it has been hypothesized that ROS play a significant role in the formation of AGEs. The targeted antioxidant mTEMPO has been shown to effectively reduce cerebral levels of age-induced RCS and AGEs in aged mice by mitigating mitochondrial ROS (Akhter et al. 2021). The exact way in which AGEs are created and why they are excessively produced in patients with diabetes are complex. In addition to the dysmetabolism caused by hyperglycemia, other factors, such as the impairment of the detoxification system (Saeed et al. 2020) and dietary exogeneous AGEs (Gill et al. 2019), also contribute to this process (Fig. 1).

## The AGE-RAGE axis and oxidative stress

The pathogenesis of diabetic complications is influenced by AGEs in three distinct ways: first, as an intracellular and extracellular protein crosslinker; second, as an accelerator of oxidative stress and inflammation; and third, as a receptor activator, which is particularly noteworthy (Serin et al. 2021). AGEs interact with multiple receptors, including scavenger receptors, Toll-like receptors (TLRs), G-protein-coupled receptors, and pattern recognition receptors (PRRs), to carry out their physiological activities (Vlassara and Striker 2011). Among these receptors, RAGE is the most extensively studied and is regarded as the primary membrane-bound receptor for AGEs. The glycosylated transmembrane receptor RAGE is a member of the immunoglobulin superfamily that consists of three domains (Bongarzone et al. 2017). These domains include an extracellular region that contains a variable (V) domain and two constant domains, C1 and C2, a transmembrane domain, and a cytoplasmic tail (Jangde et al. 2020). In addition to binding to AGEs, RAGE has been shown to bind to other ligands, such as S100 proteins, LPA, and nucleic acids (Hudson and Lippman 2018). Since RAGE can recognize analogous structural constituents within a plethora of ligands, RAGE has been classified as a PRR (Jangde et al. 2020; Syed et al. 2018). In patients with diabetes, the N-terminal V domain of RAGE serves as the primary target for AGE binding, with the adjoining C1 domain reinforcing this interaction (Kim et al. 2021a, b; Degani et al. 2017). Upon binding, a series of sustained signal transduction events are triggered by the C-terminal cytoplasmic tail, ultimately leading to the deleterious effect of AGEs. The two most well-defined pathophysiological states induced by AGEs are inflammation and oxidative stress (Twarda-Clapa et al. 2022; Garay-Sevilla et al. 2021). Our primary focus will be on how AGEs contribute to oxidative stress through their interaction with RAGE, which may serve as the initial factor for the pathology of diabetic complications.

### ROS-generating regulator: NOX4

In 2001, Wautier and colleagues conducted a study on human endothelial cells and discovered that nicotinamide adenine dinucleotide phosphate (NADPH) oxidases (NOXs) serve as pivotal downstream effectors of the AGE-RAGE signaling pathway, connecting the AGE-RAGE axis to ROS production within the cellular milieu (Wautier et al. 2001). The NOX family of NADPH oxidases is commonly accepted as one of the predominant sources of controlled ROS production (Cimmino et al. 2023). This classification system pertains to a group of transmembrane proteins that play crucial roles in facilitating electron transfer across biological membranes (Bedard and Krause 2007). Through this process, oxygen molecules are reduced to superoxide anions or hydrogen peroxide, thereby contributing to the intracellular ROS content (Jie et al. 2022; Sies and Jones 2020).

Over the past several decades, researchers have discovered seven isoforms within the NOX family, namely, NOX1, NOX2, NOX3, NOX4, NOX5, DUOX1, and DUOX2 (Vermot et al. 2021). Among these members, NOX4 has been determined to be the specific NOX isoform that responds to the interplay between AGEs and RAGE in type 2 diabetes-related nonalcoholic steatohepatitis (Dehnad et al. 2020). A different investigation involving knockout of the rat NOX gene and pharmacological inhibition of the NOX enzyme revealed that NOX4, rather than NOX1, is the principal ROS generator in diabetes-induced nephropathy (Jha et al. 2014). Thus, the potential downstream effector of the diabetic AGE-RAGE signaling pathway within the NOX family is likely NOX4.

In contrast to other NOX family members, NOX4 has unique characteristics: it can generate hydrogen peroxide as the predominant ROS product even in the absence of superoxide dismutase. This capability has been observed in vitro and in vivo (Nisimoto et al. 2014). Considering that hydrogen peroxide plays a significant role in the redox modulation of various biological processes (Sies and Jones 2020), it is rational to propose that the activation of NOX4 by the AGE-RAGE axis may trigger the supraphysiological production of hydrogen peroxide, culminating in oxidative stress. The utilization of catalpol to inhibit the AGE-RAGE-NOX4 signaling pathway has been found to be effective in inhibiting the overproduction of ROS, which in turn alleviates AGE-induced oxidative stress and protects against diabetes mellitus-induced impairment of male reproductive health (Jiao et al. 2020). This study revealed significant associations among AGE-RAGE binding, NOX4 activation, and oxidative stress development.

However, the precise mechanism through which NOX4 is stimulated upon AGE–RAGE binding remains elusive. Notably, NOX4 activation is not contingent upon any cytosolic component, as NOX4 is constitutively active (Waghela et al. 2021). Hence, the management of NOX4 activity generally involves the oversight of its transcriptional and translational mechanisms (Cimmino et al. 2023). Smad3 is a vital mediator of transforming growth factor-β1 (TGF-β1), which may act as a pivotal intermediary factor. This finding is supported by the observation that elevated glucose levels increase the phosphorylation of smad3, facilitating its translocation to the nucleus and subsequent attachment to the promoter region of NOX4 (Wang et al. 2020). The canonical TGF-β1‒Smad3 signaling pathway can be triggered by AGEs through the upregulation of TGF-β1 expression in a variety of cells (Deng et al. 2020; Huang et al. 2015). Once TGF-β1 is activated, it binds to its receptors, TGF-β receptor 1 (TGFR1) and TGF-β receptor 2 (TGFR2), which phosphorylate downstream Smad3 and Smad2. Furthermore, reports indicate that the activation of Smad3 can also be initiated by the stimulation of RAGE by AGEs through a cross-talk pathway that does not rely on TGF-β1 but rather on the ERK/p38/MAP kinase pathway in a rapid manner (Wu et al. 2022a, b; Li et al. 2004). Despite the lack of TGF-β1 or the overexpression of dominant-negative TGFR2, the activation of Smad signaling by AGEs remains unaffected, as demonstrated by the phosphorylation of ERK1/2, p38 and Smad2/3 within a time frame of 30 min (Chung et al. 2010). Upon activation, Smad2/3 is bound by Smad4, facilitating the translocation of this complex to the nucleus for the initiation of the transcription of specific genes (Meng et al. 2016). A chromatin immunoprecipitation assay identified the NOX4 promoter region as a plausible site for Smad2/3/4 binding (Wang et al. 2020). This event triggers the transcription and translation of NOX4, which is at least partially accountable for the oxidative stress induced by AGEs.

### ROS elimination regulator: Nrf2

In addition to their direct role in ROS production and oxidative stress induction, AGEs can also perturb the balance of oxidation‒reduction by impacting the antioxidative stress system. Nuclear factor E2-related factor 2 (Nrf2) has been considered the primary regulator of the cellular antioxidant response for several decades, and it is instrumental in preserving the equilibrium of the redox system. Under basal conditions, Nrf2 is inhibited by binding to Kelch-like ECH-associated protein 1 (KEAP1), which functions to mediate the ubiquitination and, ultimately, the degradation of Nrf2. Under conditions of oxidative stress, Nrf2 is stimulated to dissociate from KEAP1 and migrate to the nucleus. Upon activation, NRF2 binds with small musculoaponeurotic fibrosarcoma (sMAF) to create a heterodimer that then binds to a specific DNA sequence known as an antioxidant response element (ARE). This binding leads to the upregulation of various genes that encode antioxidant enzymes (Bellezza et al. 2018; Buendia et al. 2016; Chen et al. 2021a, b).

A well-documented observation is the compromised Nrf2-ARE antioxidant network in patients with diabetes, and this dysfunctional network is strongly correlated with the progression of diabetic complications (Subba et al. 2022; Karan et al. 2020). Hence, numerous drugs, including allopurinol (Zeng et al. 2021; Luo et al. 2020), baicalin (Ma et al. 2021), hesperetin (Chen et al. 2019) and trametenolic acid (Duan et al. 2022), have focused on the Nrf2-ARE antioxidative pathway as promising approaches for ameliorating diabetic complications. Because the excessive fructose consumption that causes AGE accumulation has the potential to hinder the activity of Nrf2 (Mastrocola 2017), the significant quantity of AGEs generated as a result of hyperglycemia could be crucial for the diabetes-related suppression of Nrf2. The initial response of hepatocytes to AGEs involves the upregulation of intracellular NRF2 within 10 min, and this upregulation may be attributed to the cellular stress response. However, prolonged exposure to AGEs for more than 200 min inhibits this effect and leads to a subsequent decrease in NRF2 levels, indicating that AGEs promote a reduction in Nrf2 (Dehnad et al. 2020).

The negative regulation of Nrf2 by AGEs may involve the glycogen synthase kinase-3 (GSK3) mechanism, which operates independently of KEAP1. Through phosphorylation, GSK3 modifies the DSGIS motif situated within the Neh6 domain of Nrf2 to form a phosphodegron. This modification leads to increased binding affinity between β-TrCP and Nrf2, which ultimately results in the recruitment of a β-TrCP-CUL1-based E3 ubiquitin ligase complex. This complex subsequently facilitates the ubiquitination and degradation of Nrf2 (He et al. 2020; Torrente and DeNicola 2022). The activation of GSK3 was detected in neuroblastoma cells stimulated with AGEs (Li et al. 2012). Furthermore, glycosylated β-amyloid (a form of AGEs, Aβ-AGEs) has greater affinity for RAGE than nonglycosylated β-amyloid (Aβ), resulting in a more significant reduction in the inhibitory phosphorylation of GSK3 (Li et al. 2013). Prolonged hyperglycemia in diabetes promotes the production of Aβ-AGEs, which may aggravate neurotoxicity and ultimately culminate in diabetic neuropathy.

However, despite serving as the primary kinase upstream of GSK3 phosphorylation, the activation or inhibition of AKT (Wang et al. 2022a, b) in response to the AGE-RAGE interaction remains controversial. Li et al. (Li et al. 2012) reported the inhibition of AKT upon AGE stimulation in human neuroblastoma SK-N-SH cells and embryonic hippocampal neurons (Li et al. 2013), resulting in the activation of GSK3. The administration of rosiglitazone to upregulate AKT can alleviate the dysfunction of endothelial progenitor cells induced by AGEs (Liang et al. 2009). However, the prevailing view is that AGEs activate AKT, which subsequently activates NF-κB, a process that is intimately linked to an inflammatory response associated with the pathogenesis of diabetic complications (Khosla et al. 2021). A recent study suggested that the use of AGEs may result in a significant reduction in insulin-mediated AKT activation in ovarian granulosa cells. Interestingly, when the cells were treated with AGEs alone, no discernible effect was observed (Diamanti-Kandarakis et al. 2016). Hence, further exploration is necessary to establish whether the activation of GSK3 by AGE-RAGE signaling is mediated by AKT or other mechanisms.

**Fig. 2:**
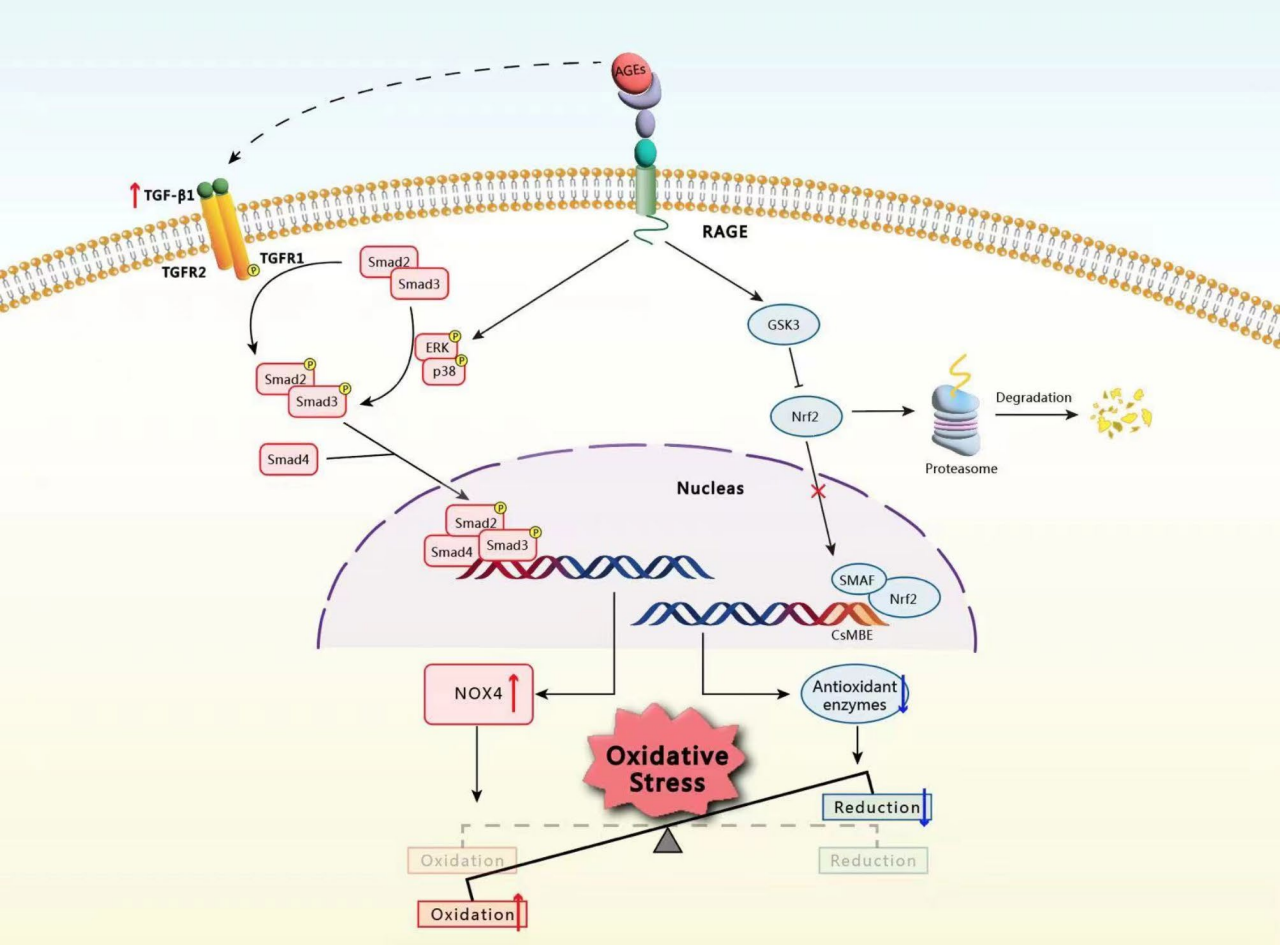
AGEs are responsible for intracellular oxidative stress by binding to RAGE and disrupting redox homeostasis. NOX4 and Nrf2 are the two targeted downstream effectors of the AGE-RAGE interaction. NOX4 is a primary contributor to the regulation of ROS generation. The canonical TGF-β1/Smad3 pathway and the noncanonical ERK/p38/MAPK/Smad3 pathway contribute to the upregulation of NOX4 by AGEs. Upon activation, Smad2/3 is bound by Smad4, facilitating the translocation of this complex to the nucleus, where it can initiate the transcription of NOX4. Nrf2 is considered the primary regulator of the cellular antioxidant response. However, AGEs can reduce the activity of Nrf2 by activating GSK3, thus diminishing its antioxidative properties. However, the mechanism by which AGEs activate GSK3 is still unclear. Augmented oxidative products, together with minimized reductive capacity, ultimately lead to oxidative stress

Under physiological conditions, NOX4 and Nrf2 work together to maintain a state of equilibrium that contributes to the maintenance of redox balance. While NOX4 can generate hydrogen peroxide, Nrf2-regulated antioxidants, such as catalase (CAT) and glutathione (Xiang et al. 2022), can effectively counteract these ROS, thereby preventing ROS accumulation. Maintaining a low and stable level of ROS that is under control can be advantageous for the growth, development, and proper functioning of cells (Lennicke and Cochemé 2021). When the harmonious interplay between NOX4 and Nrf2 is disrupted, excessive accumulation of ROS occurs, leading to oxidative stress. An abnormal increase in NOX4, coupled with a lack of Nrf2 activation in senescent myofibroblasts, leads to a persistent redox imbalance that is closely correlated with ROS accumulation and pulmonary fibrosis (Hecker et al. 2014). The restoration of the NOX4/Nrf2 redox equilibrium by *Salvia miltiorrhiza* was suggested to attenuate oxidative stress and impede the development of pulmonary fibrosis (Peng et al. 2019). As a result of the simultaneous upregulation of NOX4 expression and suppression of Nrf2 activity by AGEs, AGEs can disrupt the NOX4/Nrf2 redox balance and induce oxidative stress (Fig. 2).

## AGEs and ferroptosis

### AGEs induced oxidative stress and ferroptosis

Although hyperglycemia-induced AGEs can trigger oxidative stress, importantly, this pathological state does not directly lead to diabetic complications. Rather, the oxidative damage that ensues from this condition serves as the underlying cause of such complications. Mitochondrial dysfunction, DNA damage, protein modification, lipid peroxidation, cellular apoptosis and inflammation are all potential consequences of an overabundance of ROS (Kang and Yang 2020; Jha et al. 2016). Among these processes, lipid peroxidation has garnered increasing attention as a research topic concerning diabetes complications. When considering oxidative stress and lipid peroxidation, one must consider the regulated form of cell death, ferroptosis, as the accumulation of lipid peroxides is considered a key factor in the onset of ferroptosis (Zhang et al. 2022).

Multiple scholarly articles have described the connection between oxidative stress and ferroptosis (Yan et al. 2021; Jiang et al. 2021; Stockwell 2022; Xie et al. 2016). Unregulated lipid peroxidation due to excessive ROS levels is widely acknowledged as the critical hallmark of ferroptosis (Jiang et al. 2021; Xie et al. 2016). However, lipid peroxidation caused exclusively by oxidative stress can only be termed oxidative lipid damage (Demirci-Çekiç et al. 2022). Ferroptosis is iron-dependent and is characterized by iron overload (Chen et al. 2021a, b) and glutathione peroxidase 4 (GPX4) inactivation (Yang and Stockwell 2016).

AGEs can induce ferroptosis by disrupting iron homeostasis, promoting oxidative stress and inhibiting the antioxidative system. It is essential to maintain an appropriate level of cellular labile iron for cell survival (Zeidan et al. 2021). Supraphysiologic amounts of labile iron can promote ferroptosis through facilitation of the Fenton reaction or activation of iron-dependent enzymes (Stockwell 2022). The regulation of the labile iron pool involves the participation of two well-known proteins, namely, transferrin (Ali et al. 2022) and ferritin (Zhang et al. 2021). Transferrin is responsible for controlling the incorporation of iron, and ferritin regulates iron storage. The depletion of ferritin or the binding of transferrin to transferrin receptors can increase the levels of labile iron, thereby increasing susceptibility to ferroptosis (Jiang et al. 2021). Treatment of engineered cardiac tissues with AGEs has been shown to cause a significant increase in the level of labile iron (Wang et al. 2022a, b). One possible reason is that ferritin is downregulated under the stimulation of AGEs. Because ferritin is one of the downstream targets of Nrf2 (Sun et al. 2016) and AGE-RAGE binding promotes the degradation of Nrf2, it is conceivable that ferritin is suppressed due to the influence of AGEs. The connection between AGEs and oxidative stress has been explained in detail above. The binding of AGEs to RAGE leads to an increase in the expression of NOX4, which is a primary generator of ROS. By activating oxidative stress-induced lipid peroxidation, the upregulation of NOX4 could lead to increased ferroptosis-dependent cytotoxicity (Park et al. 2021). GPX4, together with glutathione, is generally acknowledged as the most important anti-lipid peroxidation system that protects cells from ferroptosis (Stockwell et al. 2017; Bersuker et al. 2019). By utilizing GSH, GPX4 functions as a phospholipid peroxidase that transforms harmful PUFA-PL-OOH into nonhazardous PUFA phospholipid alcohols (PUFA-PL-OH). This transformation ultimately halts the iron-dependent ROS-mediated lipid peroxidation chain reaction within the membrane and prevents lethal ferroptosis (Forcina and Dixon 2019). The biosynthesis of GSH requires an adequate supply of cysteine, which is imported by System X_c_^−^, and proper functioning of the rate-limiting enzyme glutamate-cysteine ligase (GCL) (Tang et al. 2021). Because of the regulation of System X_c_^−^ and GCL by Nrf2 (Ursini and Maiorino 2020; Dodson et al. 2019), the inhibitory effect of AGEs on Nrf2 may impede the production of GSH. This could compromise the function and activity of GPX4, potentially resulting in increased vulnerability to ferroptosis.

### AGEs induce ferroptosis and diabetic complications

These findings suggest that the accumulation of AGEs may promote ferroptosis in susceptible diabetic tissues. The induction of ferroptosis by AGEs may substantially influence the development of diabetic complications. Furthermore, increasing evidence indicates a potential link between AGE-induced ferroptosis and diabetic complications. (Table 1)

**Table 1:** Correlation between AGEs, ferroptosis and diabetic complications

Diabetic complications	Cell type/Animal model	Mechanism	Reference
Diabetic osteoporosis	Human hFOB1.19 osteoblast cell line	AGEs induce the ferroptosis of osteoblast by downregulating GPX4, SLC7A11 and GSH/GSSG levels.	(Ge et al. 2022)
Diabetic osteoporosis	C57BL/6J mice BMSCs/HFD&STZ induced C57BL/6J mice	TFNA-Cur nanoparticles could inhibit AGE-induced ferroptosis by activating the NRF2/GPX4 pathway.	(Li et al. 2024)
Diabetic periodontitis	MLOY4 murine osteocytes/HFD&STZ induced C57/BL6 mice	AGEs, together with LPS, triggers alveolar osteocyte ferroptosis via the downregulation of the SLC7A11/GPX4-axis.	(Li et al.^2023^)
Diabetic cardiomyopathy	Engineered cardiac tissue/HFD&STZ induced AMPKα2-KO mice	AGEs induce ferroptosis in ECTs by inhibiting the expression of SLC7A11 and ferritin. Sulforaphane activates the AMPK/NRF2 pathway to alleviate AGE-induced ferroptosis in cardiomyocytes.	(Wang et al. 2022a)
Diabetic kidney disease	Db/db mice	Diabetes promotes NOX-derived ROS formation in mouse kidneys. Ferroptosis might enhance DKD and damage renal tubules in diabetic models through the HIF-1a/HO-1 pathway.	(Feng et al. 2021)
Diabetic retinopathy	Human retinal endothelial cells/STZ induced C57BL/6J	High glucose-induced ZFAS1 elevation activates ferroptosis in hRECs via the ZFAS1/miR-7-5p/ACSL4 axis.	(Liu et al. 2022)
Diabetic retinopathy	ARPE-19 cells	Astragaloside-IV can inhibit high glucose induced ferroptosis via the miRNA/Sirt1/Nrf2 signaling cascade in retinal pigment epithelial cells.	(Tang et al. 2022b)
Diabetic retinopathy	ARPE-19 cells	High glucose downregulates SLC1A5 to aggravate ferroptosis of retinal pigment epithelium cells by upregulating miR-338-3p.	(Zhou et al. 2022)

Recent evidence has revealed an association between AGE-induced ferroptosis and diabetic osteoporosis (DOP). Serum AGEs isolated from patients with DOP trigger ferroptosis in hFoB1.19 osteoblast cells in vitro, leading to compromised cell viability and impaired bone formation capacity (Ge et al. 2022). In addition to osteoblasts, AGE-induced ferroptosis influences the function of bone mesenchymal stem cells (BMSCs). Activating the NRF2-GPX4 pathway via the use of curcumin to suppress AGE-induced ferroptosis in BMSCs has the potential to improve the osteogenic differentiation capacity of BMSCs in vitro and mitigate DOP in vivo (Li et al. 2024). The involvement of ferroptosis in the pathogenesis of osteocyte death has been confirmed in diabetic periodontitis, which presents characteristics of bone loss comparable to those of DOP (Wu et al. 2022a, b; Zhao et al. 2022). In the diabetic periodontal microenvironment, GPX4 and SLC7A11 are downregulated, triggering alveolar osteocyte ferroptosis and contributing to alveolar bone loss (Li et al. 2023). Notably, however, the ferroptosis of alveolar osteocytes in diabetic periodontitis is attributed to the combined influence of AGEs and lipopolysaccharide (LPS) (Li et al. 2023). Additionally, AGE-induced ferroptosis has been observed in diabetic cardiomyopathy (DCM). Exposure to AGEs leads to notable upregulation of ferroptosis markers (MDA and Ptgs2) and significant downregulation of ferroptosis protectors (GSH and SLC7A11) in engineered cardiac tissue (Wang et al. 2022a, b). The ferroptosis of cardiac cells may elucidate the mechanism by which AGEs induce cardiac remodeling and diastolic contractile dysfunction in the pathogenesis of DCM (Lou et al. 2024). Targeting the antioxidant NRF2-ARE pathway to alleviate AGE-induced ferroptosis may offer an optimal treatment approach for DCM (Wang et al. 2022a, b), mirroring its efficacy in managing DOP. These findings suggest a unified mechanism of AGE-induced ferroptosis in the progression of DOP and DCM. In addition to activating ferroptosis inhibitors, targeting AGE scavengers may also serve as a viable therapeutic strategy for treating diabetic complications. The involvement of ferroptosis in the progression of liver fibrosis in nonalcoholic steatohepatitis (NASH) with T2DM has been linked to the insufficiency of AGE receptor 1 (AGER1), an in vivo scavenger and protector of AGEs (Gong et al. 2023; Uribarri et al. 2011).Decreased AGER1 expression in hepatocytes may increase the interaction between AGEs and RAGE, potentially leading to an increased prevalence of RAGE-mediated signals (Dehnad et al. 2020) and thereby promoting the progression of ferroptosis.

The aforementioned studies have provided compelling evidence for the indispensable associations among AGEs, ferroptosis and diabetic complications. However, this association has not been clearly described in some other cases, and we can only make reasonable inferences from existing studies. Feng et al. (Feng et al. 2021) revealed that in db/db mouse kidneys, ferroptosis, rather than apoptosis, is the primary contributor to albuminuria and renal tubule injury via the HIF-1a/HO-1 pathway. This study also demonstrated that the hyperglycemic microenvironment in diabetic kidneys can increase ROS levels by upregulating NOX expression (Feng et al. 2021). Moreover, the application of the ferroptosis inhibitor ferrostatin-1 has shown potential in attenuating this process (Feng et al. 2021). Because NOXs have been identified as key players in the AGE-RAGE axis, contributing to increased oxidative stress in diabetic kidney disease (DKD) kidneys (Wu et al. 2021a, b), a potential connection between AGEs, RAGE, NOXs, ROS, and ferroptosis in DKD is plausible. Vitreous levels of AGEs have been reported to be positively correlated with the severity of diabetic retinopathy (DR) (Katagiri et al. 2018). The retina is a tissue that contains abundant PUFAs, which makes it vulnerable to oxidative stress and peroxidative challenges (Catala 2011; Ouyang et al. 2023). Recent findings have revealed that ferroptosis is involved in the pathogenesis of DR. High glucose exposure has been shown to activate the ZFAS1/miR-7-5p/ACSL4 axis in human retinal endothelial cells (hRECs), resulting in increased lipid peroxidation and the initiation of ferroptosis (Liu et al. 2022). Additional research has also identified other miRNAs, such as miR-338-3p and miR-138-5p, that are associated with high glucose-induced ferroptosis in DR (Tang et al. 2022a, b; Zhou et al. 2022). AGE has been reported to be one of the primary consequences of prolonged hyperglycemia, which causes retinal oxidative damage in diabetic individuals (Kang and Yang 2020). It is conceivable that the novel discovery of ferroptosis in DR could be linked to AGEs. Moreover, AGE-induced ferroptosis may also be predictive of other diabetic complications, including diabetic pulmonary dysfunction (Dai et al. 2023) and diabetic peripheral neuropathy (Qi et al. 2022).

Ferroptosis is highly complex and requires specific molecular mechanisms for its initiation and execution. These mechanisms include altered glucose, lipid, and amino acid metabolism; decreased antioxidative capacity; and disrupted iron regulation (Stockwell 2022). Hence, in addition to the aforementioned regulatory factors, numerous other unidentified factors require further investigation to determine their potential impact on the onset of ferroptosis triggered by AGEs in diabetic environments.

## Conclusions

There is a broad consensus on the pivotal involvement of AGEs in the onset of diabetic complications. Previous studies have generally concluded that most of the downstream effects of AGEs lead to the activation of NF-κB signaling, resulting in the release of proinflammatory molecules that exacerbate diabetic complications.

In this review, we propose a novel concept in which AGE stimulation may cause ferroptosis in diabetic tissues and organs. Moreover, we investigated the relationship between AGEs and ROS. AGE-RAGE binding increases NOX4 and suppresses Nrf2, resulting in oxidative stress. Nrf2 functions as a master regulator of redox balance and exerts significant control over iron metabolism (Shakya et al. 2023). Thus, the inhibitory effect of AGEs on Nrf2 also results in dysregulation of the cellular labile iron pool, which greatly contributes to the production of ROS via the Fenton reaction and iron-dependent enzymes. ROS then target membranous PUFA-PLs, initiating a lipid peroxidation chain reaction. This toxic reaction can be neutralized by the antioxidative system X_c_^−^/GSH/GPX4 axis, which is regulated by Nrf2. Consequently, the subsequent impacts of AGEs, such as perturbations in iron balance, increased oxidative stress, and suppression of the antioxidative system, ultimately converge on ferroptosis.

**Fig. 3:**
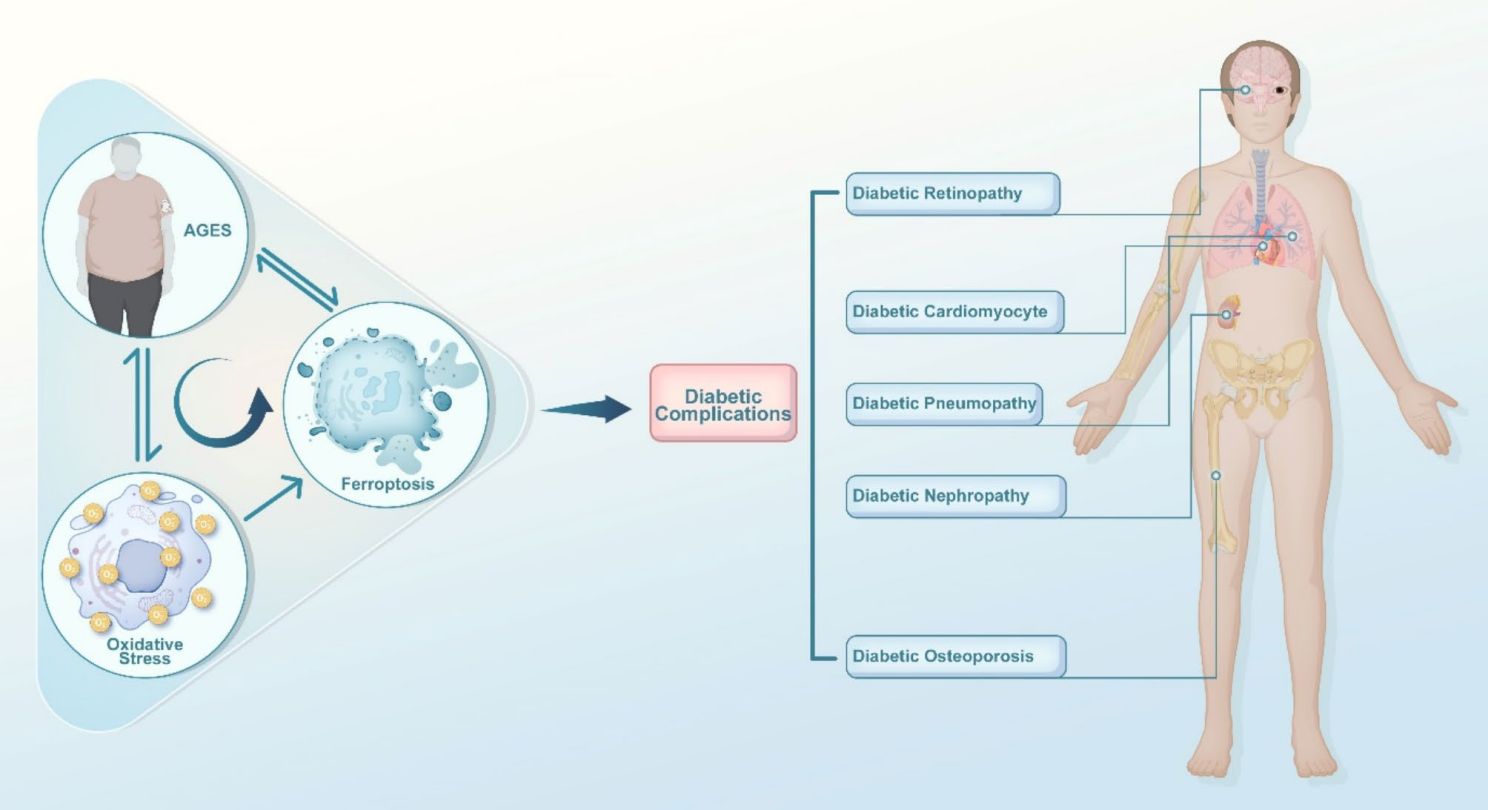
AGEs, oxidative stress and ferroptosis can be linked in a vicious cycle. AGEs and oxidative stress may interact in a mutually beneficial manner, with each being able to support the other. ROS overload renders cells highly susceptible to ferroptosis, with disrupted iron regulation and inhibited antioxidant systems caused by AGEs also being risk factors. Ultimately, the byproducts of lethal lipid peroxidation in cell membranes contribute to the RCS pool, thus promoting the production of AGEs. AGEs induce ferroptosis in conditions of diabetic neurodegeneration, diabetic cardiomyocytes, diabetic nephropathy and diabetic osteoporosis

Notably, increased oxidative stress (Mirlohi et al. 2018) and the presence of the RCS byproducts of ferroptosis increase AGE formation. As mentioned above, AGEs can be categorized into two distinct groups according to their origin: reducing sugar-derived advanced glycation end products and lipid-derived advanced lipoxidation end products. Under conditions of oxidative stress, the accumulation of ROS facilitates the promotion of glycoxidation and lipid peroxidation reactions (Moldogazieva et al. 2019). Both of these reactions produce RCSs, which are essential for the formation of AGEs. In the lethal event of ferroptosis, lipid peroxidation can directly produce MDA, 4-HNE and other RCSs, thus promoting the synthesis of AGEs. Interestingly, AGEs, oxidative stress and ferroptosis are intertwined in a vicious cycle. AGEs may be the potential cause of oxidative stress and ferroptosis; conversely, oxidative stress and ferroptosis are implicated in the production of AGEs (Fig. 3).

In conclusion, our review summarizes the mechanisms by which AGEs are overproduced in patients with diabetes and how AGE-induced oxidative stress occurs. We hypothesize that AGEs have the potential to induce ferroptosis, which could lead to diabetic complications, thus providing a concept and direction for examining the development of diabetic complications. Further exploration is necessary to understand the impact of AGEs on ferroptosis.

## Data Availability

Not applicable.

## Abbreviations

AGEs: Advanced glycation end products
RAGE: Receptor for advanced glycation end products
RCS: Reactive carbonyl species
ALEs: Advanced lipoxidation end products
AR: Aldose reductase
SDH: Sorbitol dehydrogenase
PUFA-PLs: Polyunsaturated fatty acyl tail-involving phospholipids
ROS: Reactive oxygen species
PUFA-PL-OOHs: PUFA phospholipid hydroperoxides
MDA: Malondialdehyde
4-HNE: 4-hydroxynonenal
TCA cycle: The tricarboxylic acid cycle
GAPDH: Glyceraldehyde-3-phosphate dehydrogenase
GA-3-p: Glyceraldehyde-3-phosphate
DHAP: Dihydroxyacetone phosphate
MG: Methylglyoxal
PKC: Protein kinase C
GSH: Glutathione
TLRs: Toll-like receptors
PRRs: Pattern recognition receptors
NADPH: Nicotinamide adenine dinucleotide phosphate
NOXs: NADPH oxidases
TGF-β1: Transforming growth factor-β1
TGFR1: TGF-β receptor 1
TGFR2: TGF-β receptor 2
Nrf2: Nuclear factor E2-related factor 2
KEAP1: Kelch-like ECH-associated protein 1
sMAF: Small musculoaponeurotic fibrosarcoma
ARE: Antioxidant response element
GSK3: Glycogen synthase kinase-3
GPX4: Glutathione peroxidase 4
